# Binder-Free V_2_O_5_ Cathode for High Energy Density Rechargeable Aluminum-Ion Batteries

**DOI:** 10.3390/nano10020247

**Published:** 2020-01-30

**Authors:** Achim M. Diem, Bernhard Fenk, Joachim Bill, Zaklina Burghard

**Affiliations:** 1 Institute for Materials Science, University of Stuttgart, Heisenbergstraße 3, 70569 Stuttgart, Germany; diem@imw.uni-stuttgart.de (A.M.D.); bill@imw.uni-stuttgart.de (J.B.); 2 Max-Planck-Institute for Solid State Research, Heisenbergstraße 1, 70569 Stuttgart, Germany; b.fenk@fkf.mpg.de

**Keywords:** V_2_O_5_ cathode, paper-like thin films, binder-free electrode, post-lithium-ion batteries, aluminum-ion battery

## Abstract

Nowadays, research on electrochemical storage systems moves into the direction of post-lithium-ion batteries, such as aluminum-ion batteries, and the exploration of suitable materials for such batteries. Vanadium pentoxide (V_2_O_5_) is one of the most promising host materials for the intercalation of multivalent ions. Here, we report on the fabrication of a binder-free and self-supporting V_2_O_5_ micrometer-thick paper-like electrode material and its use as the cathode for rechargeable aluminum-ion batteries. The electrical conductivity of the cathode was significantly improved by a novel in-situ and self-limiting copper migration approach into the V_2_O_5_ structure. This process takes advantage of the dissolution of Cu by the ionic liquid-based electrolyte, as well as the presence of two different accommodation sites in the nanostructured V_2_O_5_ available for aluminum-ions and the migrated Cu. Furthermore, the advanced nanostructured cathode delivered a specific discharge capacity of up to ~170 mAh g^−1^ and the reversible intercalation of Al^3+^ for more than 500 cycles with a high Coulomb efficiency reaching nearly 100%. The binder-free concept results in an energy density of 74 Wh kg^−1^, which shows improved energy density in comparison to the so far published V_2_O_5_-based cathodes. Our results provide valuable insights for the future design and development of novel binder-free and self-supporting electrodes for rechargeable multivalent metal-ion batteries associating a high energy density, cycling stability, safety and low cost.

## 1. Introduction

The rising demand for advanced energy storage systems, e.g., rechargeable metal-ion batteries, with a high energy density requires novel electrode materials and fabrication concepts to fulfill crucial requirements for their application. Those requirements include a high storage capacity and current density, as well as long-term stability, low cost and sustainability [1,2,3]. In this context, lithium-ion batteries (LIBs) have been in the focus of research due to their high energy densities and wide electrochemical potential window [4]. However, lithium is a highly reactive metal and its natural resources are quite limited [5,6]. Therefore, attention has shifted toward different mono- and multivalent ions as a substitution for lithium [7]. Among this, aluminum is especially promising as it is the third most abundant element in the earth’s crust, less hazardous and reactive compared to alkali metals. In addition, the three-electron redox couple of aluminum leads to a high theoretical gravimetric and volumetric capacity of 2980 mAh g^−1^ and 8063 mAh cm^−3^, respectively, rendering it for high energy density metal-ion batteries [8,9]. While the working principle of rechargeable aluminum-ion batteries (AIBs) has been clarified to some extent [8], the ongoing development faces several challenges. This includes identifying suitable electrolytes, current collectors and additives. Ionic liquids, such as those imidazolium-based, mixed with aluminum chloride (AlCl_3_) have shown some promise as electrolytes [10,11,12,13,14,15]. However, such electrolytes are not fully compatible with current collectors (e.g., Ni, Cu, Ta, Mo or stainless steel). In particular, stainless steel [16] and Cu [13,17,18] are dissolved by the imidazolium-based electrolytes. Furthermore, for achieving better stability of the current collectors the operating potential window needs optimization. For example, the use of Ni is limited because side reactions take place between the potentials 1.0 V and 1.5 V [17]. Similar to current collectors, binders, like polyvinylidene fluoride (PVDF), often have only limited stability against the electrolyte [12]. Finally, the role of carbon black, usually used as a conductive agent in conventionally prepared electrodes, should be taken into account. Considering all aspects, the aforementioned limitations highlight the need for suitable cell design, which includes the optimal pairing of a current collector with an electrolyte, as well as the development of self-supporting and binder-free electrodes for AIBs.

One promising class of cathode materials for AIBs are carbons—in particular, graphite and graphene—owing to their layered structure and good electrical conductivity. Lin et al. [10] reported a cathode comprised of a three-dimensional graphitic foam with a specific capacity of up to ~70 mAh g^−1^ at a current density of 4000 mA g^−1^ over more than 7000 cycles. Another study, reports that an electrode made of natural graphite flakes and PVDF exhibits a specific capacity of ~110 mAh g^−1^ at a current density of 99 mA g^−1^, and a Coulomb efficiency of ~98% [14]. Reduced graphene oxide combined with PVDF and carbon black has also been used as cathode material, yielding a capacity of 171 mAh g^−1^ at a current density of 100 mA g^−1^ [17]. Recently, Zhang and coworkers [19] could improve the capacity at high current densities of a graphene-based cathode by increasing the number of intercalation sites through the fabrication of an edge-rich graphene paper. In this manner, they reached for this cathode a specific capacity of 128 mAh g^−1^ at a current density of 2000 mA g^−1^. In general, the ability to apply high current densities for carbonaceous-based electrodes is due to the higher diffusion rate enabled by the intercalated species, chloroaluminate (AlCl_4_^−^) [20]. Carbonaceous materials do not promote the splitting of Al_2_Cl_7_^−^ into AlCl_4_^−^ and Al^3+^ at the electrode/electrolyte interface during the electrochemical reactions, thus AlCl_4_^−^ is inserted instead of Al^3+^ into the host lattice [12]. Besides the advantage of a high diffusion rate, only one electron is transferred per intercalated ion, which limits the storage capacity of such materials [8,10,14]. Therefore, active materials that provide the ability to supply Al^3+^ at the interface to the electrolyte would be beneficial to achieve a three-electron transfer resulting in higher storage capacities.

This encouraged the research on vanadium pentoxide (V_2_O_5_) as cathode material for AIBs, whose layered structure allows intercalation of various ions into the lattice, including Al^3+^ [2,3,21]. Another important advantage of V_2_O_5_ is its support to dissociate chloroaluminates into Al^3+^ at the interface between electrode and electrolyte [12]. Therefore, Al^3+^ species are available for insertion, which would offer a three-electron transfer leading to high storage capacities. A number of investigations have been done on crystalline V_2_O_5_ powder as cathodes for which storage capacities ranging between 30 and 305 mAh g^−1^ have been reported [11,13,15,22,23,24]. The relatively low values (compared to the theoretical capacity of 442 mAh g^−1^) and the wide range of reached capacities are due to the different applied current densities, used current collectors and composition of the electrodes. The latter involves binders as mechanical support for the loose V_2_O_5_ powder, while adding carbon black to the electrode solves the issue of the poor electrical conductivity. However, they do not participate in the electrochemical reactions and increase the weight of the electrode, thus reducing the gravimetric storage capacity [12]. Moreover, the additives could react with the electrolyte leading to a decline of cycling stability. Despite these disadvantages, binder-free systems have been rarely reported. One example is a cathode fabricated by the direct deposition of V_2_O_5_ on a Ni foam, which serves as mechanical support and current collector [12]. The foam structure of the cathode, as well as the close proximity of the V_2_O_5_ to Ni, resulted in an improved diffusion of the electrolyte through the network and enhanced charge exchange between V_2_O_5_ and Ni. Consequently, the electrochemical polarization is reduced compared to the binder containing electrodes. This resulted in high storage capacities of up to ~240 mAh g^−1^ at a current density of 44.2 mA g^−1^. Although the approach is very promising, it still requires Ni as mechanical support, which affects the gravimetric storage capacity of the electrode. Therefore, an approach to fabricate a self-supporting, binder-free electrode and to increase the electrical conductivity of the active material would be of great importance for the development of advanced AIBs.

For the fabrication of self-supporting electrodes, micro- and nanostructuring is a versatile strategy to improve their mechanical stability. Recently, we demonstrated that self-supporting paper-like V_2_O_5_ films are accessible through self-assembly of V_2_O_5_ nanofibers from an aqueous suspension. The films are characterized by a high mechanical stability (tensile strength up to 116 MPa and Youngs modulus of 42 GPa), which can be tailored by the nanofiber length and water content. The mechanical stability and moderate in-plane electrical conductivity of ~2 S cm^−1^ render them as a suitable binder-free cathode for metal-ion batteries, such as LIBs [25]. However, in order to take full advantage of the three-electron transfer during Al^3+^ intercalation a higher electrical conductivity in-plane and out-of-plane would be beneficial. In this respect, doping with metal-ions is one plausible approach. It was reported that the electrical conductivity of nanofiber-based V_2_O_5_ xerogels can be significantly enhanced by Cu doping resulting in the formation of Cu*_x_*V_2_O_5_ bronzes, while the layered structure of the nanofibers is preserved [26]. The increased electronic conductivity is correlated to the reduction of V^5+^ to V^4+^, thus electron hopping as transport mechanism is more dominant. The electrochemical characterization of the bronzes directly deposited on the current collectors, revealed high lithium insertion rates resulting in high storage capacities and cycling stabilities [27,28]. Therefore, Cu doping is a suitable approach to improve the electrical conductivity of V_2_O_5_ thin films, which would render them as a promising binder-free and self-supporting cathode material for AIBs.

Here, we report the use of a binder-free and self-supporting cathode, for Al^3+^ intercalation, comprised of V_2_O_5_ nanofibers in the form of micrometer-thick self-supporting thin films. The cathode is fabricated via a self-assembly approach resulting in an aligned and layered structure of high mechanical stability [25]. The optimized cell setup and testing parameters included a suitable current collector and a potential window that avoids undesired side reactions with the electrolyte. A Cu doping process was employed to enhance the electrical conductivity of the V_2_O_5_ cathode. Such cathodes enable the reversible intercalation of Al^3+^ for more than 500 cycles with a high specific discharge capacity of up to ~170 mAh g^−1^, high Coulomb efficiencies and an energy density of 74 Wh kg^−1^. Our findings provide novel guidelines and insights to overcome the faced challenges and proceed with the development of future AIBs.

## 2. Materials and Methods

*Synthesis of V_2_O_5_ dispersion:* The V_2_O_5_ nanofibers are synthesized by the mixing of ammonium meta-vanadate (1 g, Fluka, Munich, Germany) and an acidic ion-exchanger (10 g, Dowex 50WX8 50-100, Alfa Aesar, Kandel, Germany) in deionized water (200 mL) [21], which is heated for 10 min in an 80 °C oil bath. After slowly cooling down to room temperature, the dispersion of the nanofibers was aged for 42 days.

*Fabrication of V_2_O_5_ cathodes:* Silicon (100) wafers (p-type, Wacker, Sitronic AG, Munich, Germany) were used as substrates, which were cleaned with chloroform, acetone and ethanol each for 10 min in an ultrasonic bath and subsequently dried in a nitrogen flow. These Si wafers were placed in a petri dish and a 1:1 dilution (9 mL) of the V_2_O_5_ nanofiber dispersion and deionized water was added. After the complete evaporation of the water under ambient conditions, the V_2_O_5_ film was removed from the Si wafer in a water bath to obtain the self-supporting paper-like film. The self-supporting papers were dried in a two-step procedure in a climatic chamber (VC 7018, Vötsch, Industrietechnik GmbH, Balingen, Germany). The first step involved increasing the humidity from 50% to 80% and temperature from 25 °C to 40 °C in 15 min. The humidity, after one hour, was decreased to 20% within 10 h, while the temperature was kept constant for 15 h. The humidity of 20% was held for 4.5 h. Finally, the temperature was reduced to room temperature. The second step consisted of a temperature increase from 25 °C to 100 °C in 30 min, which was held for two hours followed by a temperature reduction to 25 °C in 30 min.

*Microstructural characterization:* For microstructural investigations, scanning electron microscopy (SEM, Zeiss Ultra 55, Zeiss GmbH, Oberkochen, Germany) and transmission electron microscopy (TEM, Philips CM200-FEG, Thermo Fisher, Hillsboro, OR, USA) equipped with an EDX (CM200, EDAX, Thermo Fisher, Hillsboro, OR, USA) at 200 kV system also for selected area diffraction (SAD) and atomic force microscopy (AFM, MultiMode 8, Nanoscope V, Bruker, Santa Barbara, CA, USA), were used. The TEM lamellae were prepared by an SEM-FIB system (1540 XB CrossBeam, Zeiss GmbH, Oberkochen, Germany). X-ray diffraction was performed by a PXRD (Rigaku Smartlab, Neu-Isenburg, Germany) in Bragg–Brentano geometry using copper K_α_ radiation, an acceleration voltage of 40 kV and a current of 30 mA in the range of 5°–40° with 0.02° as step size.

*Electrical characterization:* The out-of-plane electrical conductivity was determined via a two-point configuration (SourceMeter 2400, Keithley, Cleveland, Ohio, USA).

*Electrochemical characterization:* For the cell assembly in an argon-filled glovebox (Labmaster SP, MBraun, Garching, Germany) Swagelok^TM^ union connections made of PTFE were used with stainless steel contacts. The contacts were protected from the electrolyte either by a commercial PE foil or by a Ta plate, which was glued with silver paste (Plano GmbH, Wetzlar, Germany) on top of the contact. The remaining steel part was coated with clear lacquer (Swingcolor, BAHAG AG, Mannheim, Germany) (Figure S6, Supporting Information). The V_2_O_5_ cathode was sputtered from both sides with a gold layer (35 nm, SCD 040, Balzers Union) to reduce the contact resistance toward the current collector. As the doping source, a 25 µm thick Cu foil (purity 99.98%, Sigma-Aldrich Chemie GmbH, Taufkirchen, Germany) was used with a diameter 1/3 smaller than the diameter of the V_2_O_5_ cathode. An 8 µm thick Al foil (purity 99%, Sigma Aldrich) was used as anode and 6 layers of glass fiber membrane (Grade 934-AH, Whatman, Sigma Aldrich) as separator, and 1-ethyl-3-methylimidazolium chloride mixed with aluminum chloride in the ratio of 1:1.5 (IoLiTec Ionic Liquids Technologies, Heilbronn, Germany) was set as electrolyte. Galvanostatic charge/discharge tests were carried out with 25, 50, 100, 200 and 500 mA g^−1^ as current densities in the voltage range of 0.2–1.1 V. Therefore, the cells were held for at least 2 h in open-circuit conditions.h in open circuit voltage (OCV) condition, followed by a pre-cycling for 50 cycles with a current rate of 1000 mA g^−1^. Cyclic voltammetry was performed in the voltage window of 0.02–1.5 V and 0.2–1.1 V, respectively, with a sweep rate of 0.1 mV s^−1^. Electrochemical impedance spectroscopy was carried out with a frequency range of 0.1–10^6^ Hz and an amplitude of 10 mV. The determined EIS data were fitted using an equivalent circuit model (*R*_1_ + *R*_2_/*C*_2_ + *W*_d_) including *R*_1_ as contact resistance, *R*_2_ as bulk resistance, *C*_2_ the bulk capacitance and *W*_d_ as Warburg element. The stability tests of Ta and Cu were carried out by CV in potential windows 0.02–2.5 V and 0.2–1.1 V with scan rates of 1 mV s^−1^ and 0.1 mV s^−1^. All measurements were performed on electrochemical test stations (VSP300, Biologic and 660C, CH Instruments, Seyssinet Pariset, France).

## 3. Results and Discussion

A binder-free and self-supporting cathode for AIBs was fabricated from nanocrystalline V_2_O_5_ nanofibers. The nanofibers are obtained via the sol–gel method and grow via an anisotropic polycondensation reaction resulting in a rectangular cross-section of the nanofiber with a height of ~1.5 nm and 10–20 nm in width [21]. They are composed of a bilayered chain-like arrangement of VO_5_ polyhedrons, separated by a water molecule layer that ensures an interlayer distance of up to 1.77 nm [21,29]. Such distance is large enough to accommodate ions or even small molecules [2,21]. The length of the nanofibers is in the range of several micrometers and can be adjusted by the storage temperature and the age of the nanofibers solution [25,30]. In this study, the used V_2_O_5_ nanofibers have a length of approximately 2 µm.

**Figure 1:**
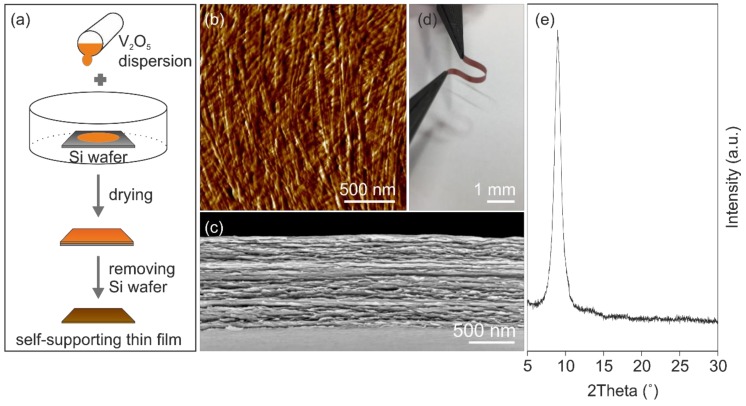
(**a**) Schematic illustration of vanadium pentoxide (V_2_O_5_) thin film preparation. A Si wafer is placed in a beaker that is filled up with the V_2_O_5_ dispersion. Drying at ambient conditions and removing the Si wafer from the V_2_O_5_ thin film results in the self-supporting V_2_O_5_ paper-like thin film. (**b**) AFM image of the paper-like surface showing the nanofiber alignment. (**c**) SEM image of the V_2_O_5_ paper revealing a layered structure. (**d**) Shaped V_2_O_5_ paper showing the flexibility. (**e**) XRD pattern of the V_2_O_5_ paper.

The presence of functional surface groups, e.g., hydroxyl- and oxo-groups attached to the nanofibers promote their self-assembly via hydrogen bond formation into self-supporting micrometer-thick paper-like thin film (Figure 1a). The self-assembly results in a good in-plane alignment of the nanofibers (Figure 1b), within an ordered layered structure as the cross-section reveals, shown in Figure 1c. This highly ordered structure, made of bendable nanofibers caused by their high aspect ratio, leads to mechanical flexibility of the films that allows shaping them into any desired form (Figure 1d). Furthermore, the mechanical properties of the films can be tailored by thermal treatment, which supports the formation of oxygen bridges between the nanofibers and thus reinforcing the entire structure [31]. Therefore, the paper-like thin films were thermally treated at 100 °C. The annealing temperature of 100 °C was chosen in order to achieve a compromise between water content and preserving the structural integrity of the nanofibers. Specifically, the presence of water is important, since water molecules keep a necessary distance between the layers. This distance is of great importance for ion insertion. Furthermore, the water molecules could shield the high charges of intercalated Al^3+^ leading to a faster shuttling of the ions into the host lattice, as proven for the co-intercalation of water and magnesium ions [32]. The hydration state of V_2_O_5_ ∙ *n* H_2_O defines the interlayer distance of the V_2_O_5_ bilayer [21], which was investigated by X-ray diffraction (XRD). The XRD pattern (Figure 1e) of the annealed self-supporting paper-like thin film reveals one clear reflection at 8.98°, which correlates to an interlayer distance of 0.984 nm and the hydration state *n* = ~1.15. For this work, we used such thermally treated films as self-supporting and binder-free cathodes for AIBs. A defined micro- and nanostructure of such electrodes should provide accessible intercalation sites and short diffusion paths for the Al^3+^, thus positively contribute to the kinetic properties of the electrode.

It was shown that the electrical conductivity of V_2_O_5_ xerogels comprised of nanofibers doped with silver or copper is enhanced by more than two orders of magnitude [26]. Accordingly, we improve the conductivity of our V_2_O_5_ cathode material by a one-step doping approach. This approach takes advantage of the synergy of the dissolution of Cu by the imidazolium-based ionic liquid electrolyte [10,12,14], and the Cu migration into the V_2_O_5_ cathode. Based on the fact that Cu corrosion in this electrolyte takes place around 1.5 V vs. Al/Al^3+^ [18], we performed electrochemical testing with 1.5 V as cut-off potential, insuring the Cu dissolution and thereby the doping approach. In conclusion, the one-step approach is comprised of the simultaneous doping of the cathode by Cu and electrochemical intercalation of the Al^3+^. Our binder-free and self-supporting V_2_O_5_ cathode was placed on a copper foil, which acts as a dopant source. Aluminum foil was employed as the counter electrode and glass fiber membranes as separator. A mixture of 1-ethyl-3-methylimidazolium chloride ([EMIM]Cl]) and aluminum chloride (AlCl_3_) was used as electrolyte at a ratio of 1:1.5, which guarantees the presence of AlCl_4_^−^ and Al_2_Cl_7_^−^ complexes [10]. For our studies, we used a Swagelok-type cell with stainless steel contacts, protected by commercial polyethylene (PE) foil to avoid reactions with the electrolyte.

**Figure 2:**
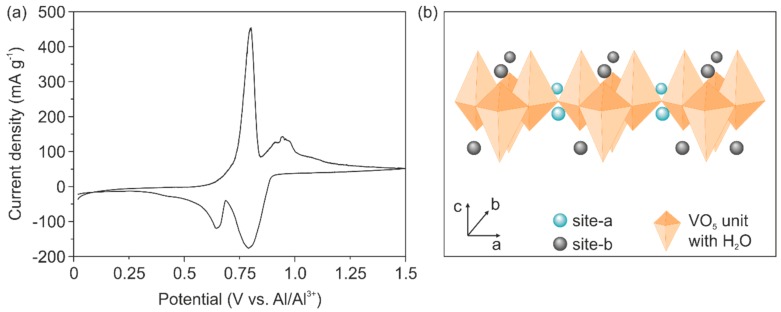
(**a**) Cyclic voltammetry curve of the second cycle at a scan rate of 0.1 mV s^−1^ revealing two de-/intercalation potentials. (**b**) Schematic illustration of the two different intercalation sites near the planar oxygen atom (site-a) and close to the apical oxygen atom (site-b) of the VO_5_ units.

Cyclic voltammetry (CV) measurements reveal reversible ion intercalation, as shown in Figure 2a. This result indicates that the electrical conductivity of the cathode is enhanced by the Cu doping and is high enough to support Al^3+^ intercalation. Specifically, two distinct intercalation potentials at 0.79 V and 0.64 V and de-intercalation potentials of 0.80 V and 0.94 V are visible. The intercalation potentials are higher than those reported for binder-free Ni-V_2_O_5_ electrodes (0.6 V), revealing the impact of reduced electrochemical polarization due to the absence of the binders [12]. Moreover, the two observed de-/intercalation peaks might be correlated to the two different intercalation sites found for V_2_O_5_ structures, reported for the intercalation of Li^+^ and Mg^2+^ [33]. In particular, the higher intercalation potential is correlated to the insertion of the ions at inner layer sites of the VO_5_ unit (site-a in Figure 2b), which is in the vicinity of the square planar oxygen atoms. Likewise, the lower intercalation potential is correlated to the insertion of the ions at places close to the apical oxygen atom of the VO_5_ unit (site-b in Figure 2b). The CV curve further reveals a stronger peak at higher intercalation potential, indicating that the inserted Al^3+^ prefers intercalation on the inner layer sites (site-a). An explanation for this phenomenon could be that this site offers four-fold coordination by the oxygen atoms thus a better charge accommodation of the intercalated ion [34].

**Figure 3:**
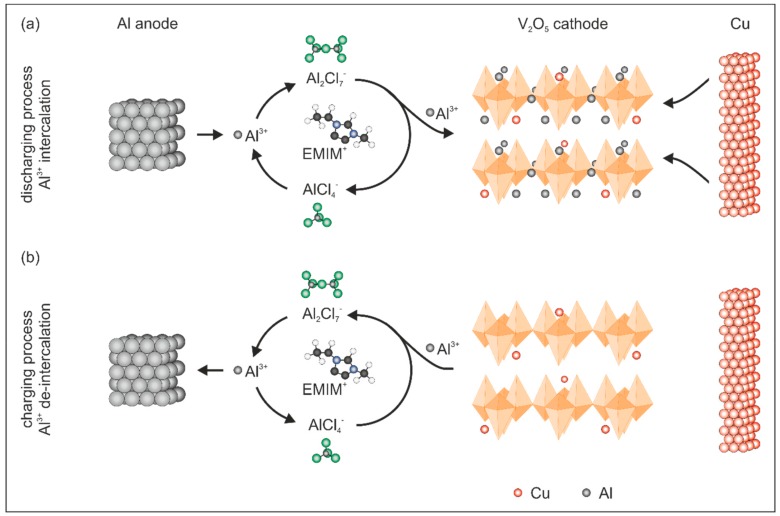
Simplified schematic representation of the occurring reactions during the discharge and charge process in the Cu doped V_2_O_5_ cathode. (**a**) In the discharge process, Al^3+^ are electrochemically stripped from the Al anode and are intercalated into the V_2_O_5_ cathode via an intermediate Al_2_Cl_7_^−^ complex. (**b**) During the charge process, Al^3+^ is de-intercalated from the V_2_O_5_ cathode forming the intermediate Al_2_Cl_7_^−^ complex and metallic Al is deposited on the anode.

A simplified reaction mechanism of the de-/intercalation processes in V_2_O_5_ is presented in Figure 3. In general, Al^3+^ is stripped from the Al anode during the discharge process (Figure 3a) and forms with AlCl_4_^−^ and the larger complex Al_2_Cl_7_^−^, which splits into AlCl_4_^−^ and Al^3+^ at the interface between the electrolyte and the cathode. Thereby, Al^3+^ is available and subsequently intercalated into the V_2_O_5_ host lattice. The following simplified reactions occur during the intercalation process:

Anode: Al − 3 e^−^ → Al^3+^, (1)

Cathode: V_2_O_5_ + *x* Al^3+^ + 3 e^−^ → Al*_x_*V_2_O_5_, (2)

During the charging process, the de-intercalation of Al^3+^ occurs (Figure 3b) leading to the formation of an Al_2_Cl_7_^−^ complex at the interface between the electrolyte and the cathode. The formed Al_2_Cl_7_^−^ complex dissociates on the anode side to AlCl_4_^−^ and metallic Al. The latter is subsequently deposited on the Al anode. Therefore, the de-intercalation reactions occur according to:

Anode: 4 Al_2_Cl_7_^−^ + 3 e^−^ → Al + 7 AlCl_4_^−^, (3)

Cathode: Al*_x_*V_2_O_5_ − 3 e^−^ → V_2_O_5_ + *x* Al^3+^, (4)

The reversibility of the stripping (Equation (1)) and deposition (Equation (3)) reaction is directly connected to the ratio of [EMIM]Cl to AlCl_3_ and the resulting acidity of the electrolyte. In order to achieve the required acidity, the ratio has to be in the range between 1:1 and 1:2 that the aluminum stripping and deposition is guaranteed [23].

Based on the fact that Cu is dissolved by the electrolyte, as indicated in Figure 3b, we conclude that Cu doping occurs during the electrochemical processes in the V_2_O_5_ cathode. To this end, the migrated Cu can react irreversibly with V_2_O_5_ to a Cu*_x_*V_2_O_5_ bronze, similar to the chemically doped Cu*_x_*V_2_O_5_ bronze [26]. To characterize the impact of Cu doping on the structure and morphology of our cathode, we performed ex-situ XRD analysis of the cathodes after the second and eighth CV cycles. The obtained XRD patterns (Figure S1, Supporting Information) reveal that the main reflection around 9.00°, observed for the pristine cathode (Figure 1d), is also present for the Cu doped V_2_O_5_ cathode after both CV measurements. This indicates that the structural integrity, e.g., V_2_O_5_ sheet stacking, of our cathode is preserved. However, a small shift toward higher 2θ values (from 8.98° to 9.44°) reveals that the distance between the V_2_O_5_ sheets is reduced. The reason might be the attraction between the positive Cu ions and the negatively charged V_2_O_5_ sheets [26]. In addition, the chemical stability of the V_2_O_5_ paper-like electrode in the used ionic liquid-based electrolyte is shown by the unchanged XRD patterns of the electrode after cycling. If any reaction would take place, then the cycling of the electrodes would not be possible. To visualize the electrode stability, digital images of the V_2_O_5_ immersed in the ionic liquid-based electrolyte after 0 min, 30 min, 60 min and 20 h were taken (Figure S2, Supporting Information).

**Figure 4:**
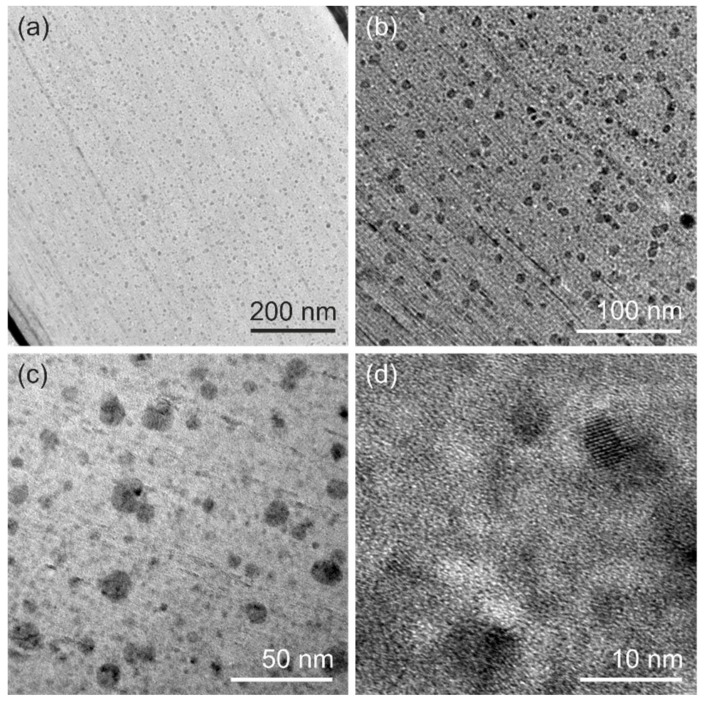
Images obtained during ex-situ TEM investigation of the cross-section of a cathode cycled eight times during CV investigations. An overview of the investigated cross-section is shown in image (**a**). The black regions correspond to the Cu-rich precipitations, which are homogeneously distributed over the complete sample. The images (**b**–**d**) are the higher magnification spots from the image (**a**).

To further investigate the influence of the migrated Cu on the morphology of the V_2_O_5_ cathode, we conducted ex-situ transmission electron microscopy (TEM) on the cathode after discharge cycles. The ex-situ TEM investigation of a cathode after two cycles of CV showed the typical layered V_2_O_5_ sheet stacking (Figure S3, Supporting Information). Contrary to that, the TEM micrographs of a cathode cycled for eight CV cycles (Figure 4a) revealed the regular stacking of V_2_O_5_ sheets and homogenously distributed precipitations (dark regions in Figure 4) over the complete cross-section. The precipitates are spherically shaped (Figure 4b) with an average diameter of 9.18 ± 3.30 nm (Figure 4c) and have crystalline lattice planes with a lattice distance in the range of 0.2–0.3 nm (Figure 4d). Such precipitates are assumed to nucleate at voids or places of imperfect alignment of the nanofibers, or at the connection points between nanofibers, which serve as heterogeneous nucleation spots for the forming precipitates. Their local chemical composition is determined by energy-dispersive X-ray spectroscopy (EDX). A high amount of vanadium and copper detected by elemental mapping relates to the Cu migrations into the V_2_O_5_ cathode. A quantitative analysis (Table S1, Supporting Information) revealed that the V_2_O_5_ matrix (bright region in Figure 4) exhibits a nearly 1:1 ratio of vanadium to copper. In contrast, the precipitates show a significantly higher amount of copper than vanadium with a vanadium-to-copper ratio of 3:4. Thus, we conclude that the migrating Cu results in doping of our V_2_O_5_ cathode and forms Cu-enriched precipitates (see Figures S4 and S5, Supporting Information), which are evenly distributed over the entire cathode.

Our conclusion was corroborated by the determination of the electrical conductivity, which is significantly enhanced by the Cu doping [26]. The electrical conductivity of our V_2_O_5_ cathodes is comprised of an electronic and ionic contribution, specifically electron hopping along the vanadium centers (V^5+^ and V^4+^) and proton diffusion alongside the nanofiber’s surface, respectively [21,25]. The in-plane electrical conductivity in the range of 2 S cm^−1^ [25,31], which is parallel to the V_2_O_5_ sheets, is approximately five orders higher than the out-of-plane electrical conductivity [31]. In the present work, we investigated the out-of-plane conductivity of our V_2_O_5_ cathodes, as it is the limiting factor for electrochemical testing. We determined a value of 0.16 ∙ 10^−6^ S cm^−1^ for the cathode before CV cycling, whereas after CV cycles two and eight, the electrical conductivity was 0.07 ∙ 10^−2^ S cm^−1^ and 0.16 ∙ 10^−2^ S cm^−1^, respectively. This significant increase by four orders of magnitude verifies several facts. First, the Cu doping is accompanied by an enhancement of the electrical conductivity. Second, that already after two CV cycles a certain amount of Cu is migrated, although not visible by TEM. Finally, the two-fold increase in conductivity of the cathode after eight cycles compared to the values after two cycles underline the significant impact of the Cu doping on the electrical conductivity.

Based on all these findings, we further optimized our cell setup to avoid all undesirable side reactions. In this respect, Ta plates were fixed on the stainless steel contacts, which were subsequently coated with a clear lacquer to prevent any reaction between the stainless steel and the electrolyte (see Figure S6, Supporting Information). Furthermore, we determined the optimal voltage window, where no side reactions attributed to the cell setup occur. For that purpose, CV was performed in a Ta vs. Ta plate configuration in the voltage range of 0.02–2.5 V with a scan rate of 1 mV s^−1^ (Figure S7, Supporting Information). The CV curve revealed that side reactions start at ~1.5 V, attributing to either the Ta plate or the clear lacquer corrosion. In addition, some minor side reactions were observed below 0.2 V. In addition, it was important to determine at which potential Cu dissolution starts in order to use Cu as a doping source. Therefore, we performed electrochemical measurements for the bare Cu foil as the cathode (Figure S8, Supporting Information). We observed weak peaks on the CV curves for the potentials above 1.0 V, which we correlated to the side reactions or Cu corrosion. Accordingly, the potential window from 0.2 V to 1.1 V was set for the electrochemical investigations. It supports the required dissolution of Cu, circumvents severe Cu corrosion and assures the stability of the current collectors. From the electrochemical impedance spectroscopy (EIS) measured on the Ta vs. Ta plate configuration, the aluminum-ion conductivity was estimated to be 5.54 × 10^−3^ S cm^−1^, which is comparable to other reported electrolytes [35,36].

**Figure 5:**
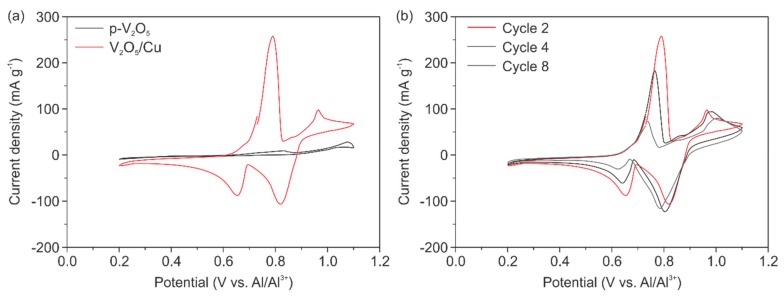
Cyclic voltammetry curves at a scan rate of 0.1 mV s^−1^ of (**a**) second cycles for p-V_2_O_5_ and V_2_O_5_/Cu revealing the importance of the migrated Cu, as well as for (**b**) cycles 2, 4 and 8 of V_2_O_5_/Cu.

This optimized cell setup was used for electrochemical characterization of the pure V_2_O_5_ cathode (p-V_2_O_5_) as a reference and the Cu doped V_2_O_5_ cathode (V_2_O_5_/Cu). The source for Cu was a micrometer-thick Cu foil placed between the cathode and Ta plate. The respective CV curves obtained after two cycles for both cathodes are presented in Figure 5a. The CV curve of p-V_2_O_5_ reveals no electrochemical performance at all, due to the absence of any intercalation and de-intercalation peaks. On the contrary, the CV curve of V_2_O_5_/Cu reveals two clear intercalation peaks at potentials of 0.82 V and 0.65 V and two de-intercalation peaks at 0.79 V and 0.96 V. This underlines the importance of the Cu presence on the electrochemical performance of the cathode, which is only due to the enhanced electrical conductivity of the cathode. To prove that the Cu is not electrochemically active within the used voltage window, additional tests were done. Corresponding CV and galvanostatic charge/discharge measurements with bare Cu as the cathode are presented in Figures S8–S10 (Supporting Information). Likewise, the potentials peaks also indicate that the two intercalation positions are occupied by the ions, as explained before site-a and site-b. Here, it should be noted that we observed two de-/intercalation potentials, while in all other reported works only one potential is stated. In this respect, the intercalation potential at 0.65 V is similar to the reported insertion potentials for various crystalline V_2_O_5_-based AIBs [11,12,13,23], while the second intercalation potential at 0.82 V is comparable to the observed insertion potential of 0.8 V, reported by Chiku et al. [15], for the cathode made of an amorphous V_2_O_5_/C composite mixed with carbon black and polytetrafluoroethylene (PTFE). The presence of the two available intercalation sites, and hence two observed peaks for our V_2_O_5_ paper-like thin film, conforms with the morphology comprised of crystalline nanofibers and their amorphous arrangement. Therefore, we could conclude, that the intercalation site-a (0.82 V) is attributed to the amorphous fraction and site-b (0.65 V) to the nanofibers. It would be of great interest to investigate the relationship between structure and intercalation sites in more detail to pronounce the advantage of such self-assembled binder-free electrodes even more.

Furthermore, we observed a change of the position of the inter- and de-intercalation potentials after the fourth and eighth cycles for V_2_O_5_/Cu, as shown in Figure 4b. The peak positions are slightly shifted to lower potentials indicating that less energy for ion diffusion through the material is required. This can be explained by the fact that the interfaces between the cathode material and the electrolyte are generated and subsequently stabilized by the ongoing inter- and de-intercalation processes. The CV curves in Figure 5b reveal a decrease of the current density for the intercalation potential of ~0.65 V and the de-intercalation potential of ~0.79 V. These potentials are referred to as intercalation site-b close to the apical oxygen atom. The same place was reported to be favorable as an accommodation site for the Cu used to dope V_2_O_5_ xerogels [34]. Thus, CV curves reveal the irreversible incorporation of Cu into our cathode during electrochemical cycling. In respect to that, our results show that Cu migrates into site-b, thus fewer intercalation sites for Al^3+^ are available. In contrast to that, the peak intensity for the intercalation potential of 0.82 V and de-intercalation potential of 0.96 V (corresponding to the site-a) is unchanged. This indicates the reversible Al^3+^ insertion and that the intercalation at site-a, close to the planar oxygen atom of the VO_5_ units, is favorable.

**Figure 6:**
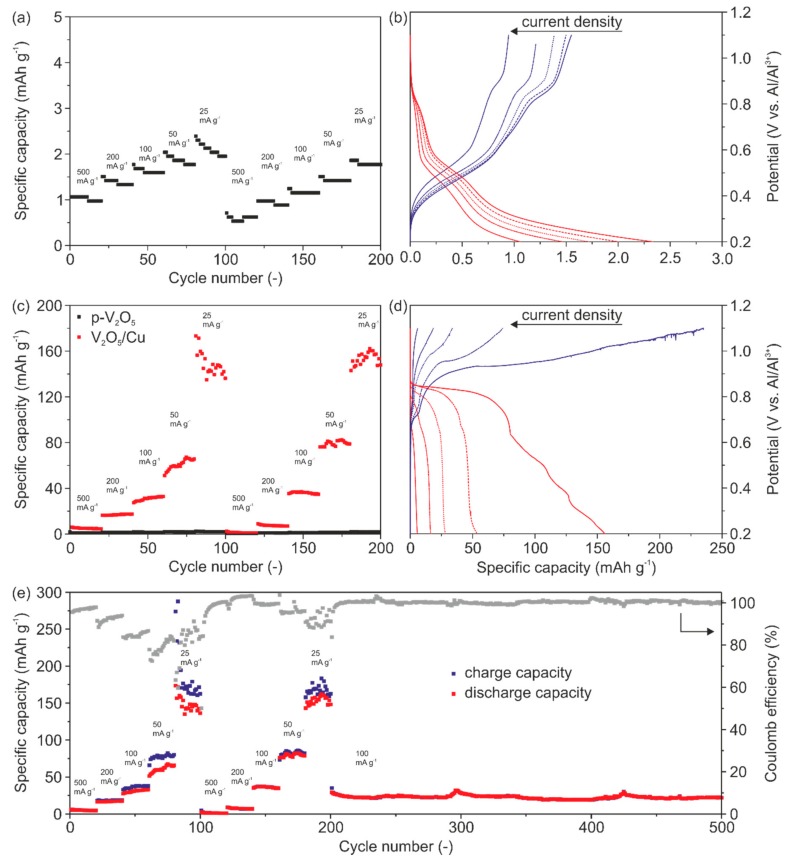
Galvanostatic charge and discharge measurements with various current densities, as displayed in the diagrams. Specific storage capacity as a function of the cycle number. (**a**) Specific discharge capacity of p-V_2_O_5_. (**b**) Potential vs. specific capacity plot for p-V_2_O_5_. (**c**) Specific discharge capacity of p-V_2_O_5_ and V_2_O_5_/Cu. (**d**) Potential vs. specific capacity plot for the V_2_O_5_/Cu. (**e**) Charging/discharging capacity and Coulomb efficiency of the V_2_O_5_/Cu cathode.

The CV measurements were complemented by galvanostatic charge/discharge tests to determine the specific storage capacity of our binder-free and self-supporting V_2_O_5_ cathodes. Figure 6a represents the specific discharge capacity as a function of cycle numbers for p-V_2_O_5_ revealing a very low discharge capacity of up to ~2.3 mAh g^−1^ at the lowest applied current density of 25 mA g^−1^. Low discharge capacities are found for all current rates and no clear intercalation and de-intercalation plateaus can be seen (Figure 6b). These results verify that p-V_2_O_5_ shows no electrochemical performance at all, as already indicated by the CV measurements (Figure 5a). The low discharge capacity compared to other reported V_2_O_5_-based cathodes, with carbon black as a conductive agent, is attributed to the very low electrical conductivity of p-V_2_O_5_, made only of active material.

This becomes even more obvious by the comparison of the specific storage capacities of p-V_2_O_5_ and V_2_O_5_/Cu cathodes (Figure 6c). Prior to all charge/discharge experiments, the cells undergo a pre-cycling of 50 cycles at a current rate of 1000 mA g^−1^. This pre-cycling for V_2_O_5_/Cu was used to induce the Cu migration into the V_2_O_5_ cathode (Figure S11, Supporting Information). In order to further investigate the impact of the migrated Cu on the electrical conductivity, the pre-cycling was coupled with EIS measurements. Therefore, for the first 20 cycles and cycles 30, 40 and 50 EIS was performed in the fully charged and discharged state. The gained EIS data were fitted with a respective equivalent circuit model and the bulk resistance was determined (Figure S12, Supporting Information). The resistance in the fully discharged state is not influenced by the pre-cycling and stays constant. However, in the fully charged state, where Al^3+^ is absent, the resistance shows a continuous decrease. This decrease of resistance or increase in conductivity is attributed to the migrated Cu.

For all investigated current densities (Figure 6c) much higher discharge capacities for V_2_O_5_/Cu were determined. The corresponding capacity profiles for the first cycles as a function of the current density are shown in Figure 6d. In particular, we determined specific discharge capacities of 6 mAh g^−1^, 16 mAh g^−1^, 27 mAh g^−1^, 53 mAh g^−1^ and 155 mAh g^−1^ with the corresponding Coulomb efficiencies of 99%, 88%, 81%, 72% and 65% at current densities of 500 mA g^−1^, 200 mA g^−1^, 100 mA g^−1^, 50 mA g^−1^ and 25 mA g^−1^. Figure 6e shows the specific charge, discharge capacities and Coulomb efficiency over cycles for the applied current densities. The initial Coulomb efficiency (ICE) is about 95% at a current density of 500 mA g^−1^. The low Coulomb efficiencies and the visible potential drops in Figure 6d indicate minor side reactions. The side reactions at this potential are caused neither by the electrolyte nor by the V_2_O_5_ electrode electrochemical stability (Figure S2, Supporting Information). However, they are mainly attributed to Cu dissolution. These side reactions are more pronounced during the charging process at low current densities thus resulting in a decrease of the Coulomb efficiency to 70%.

The diagram in Figure 6e further reveals that the storage capacities and Coulomb efficiencies are stabilized over time by comparing the first and second blocks of the five current densities. Moreover, it can be seen that our cathode delivers a constant discharge capacity of ~25 mAh g^−1^ at a current density of 100 mA g^−1^. Furthermore, the Coulomb efficiency close to 100% underlines the high reversibility toward Al^3+^ intercalation and cycling stability over 300 cycles. The latter is attributed to the ability of the cathode’s microstructure to effectively accommodate the stresses upon cycling.

The impact of the migrated Cu on the electrical conductivity of the cathodes is evident through the discharge capacities of p-V_2_O_5_ and V_2_O_5_/Cu. Specifically, for the highest current density, the discharge capacity of V_2_O_5_/Cu is about 150% higher than for p-V_2_O_5_. We assume that the Cu migration in V_2_O_5_/Cu is a self-limiting process. By cycling, a plateau is observed, referring to saturation of migrated Cu within the cathode. The saturation is due to the irreversible migration of Cu, thus the number of available intercalation sites is reduced. Furthermore, the Cu saturation is accompanied by the enhancement of the electrical conductivity of the cathode. This enables an enhanced de-/intercalation of Al^3+^ of up to 0.38 mole aluminum per V_2_O_5_ unit. Moreover, optimization of the dopant source would be beneficial to achieve a compromise between the amount of migrated Cu, the resulting enhanced electrical conductivity and availability of Al^3+^ intercalation sites.

To validate our fabrication concept of a binder-free and self-supporting V_2_O_5_ cathode to other cathode materials for AIB, the energy density was calculated and summarized in Table S2 (Supporting Information). We determined an energy density for V_2_O_5_/Cu of 74 Wh kg^−1^ (173 mAh g^−1^ at 25 mA g^−1^), which is comparable to carbon-based electrode materials [10,14,17,19]. Furthermore, the energy density of our V_2_O_5_/Cu is superior to the values of other reported V_2_O_5_-based cathodes [11,12,13,15,22,23]. This improvement is partly attributed to the omission of binders during the fabrication of the electrodes. In addition, these electrodes, exclusively made of active material, are assembled in a uniform aligned microstructure. The advantage of this microstructure is its improved accessibility for ions throughout the entire electrode. Therefore, the Cu migration and the Al^3+^ de-/intercalation are facilitated, which strongly enhances the electrical conductivity and the storage capacity, respectively. These findings provide a big leap into the development of novel advanced electrodes for AIB.

## 4. Conclusions

We successfully obtained a self-supporting and binder-free paper-like thin film comprised of V_2_O_5_ nanofibers doped with Cu to enable its application as cathode material for AIBs. The Cu doping relies on the dissolution of Cu by the ionic liquid-based electrolyte and the subsequent Cu migration into the V_2_O_5_ films. While the Cu migration notably increases the electrical conductivity of the films, their high mechanical stability and flexibility that originates from their regular, hierarchical layer structure is effectively preserved after Cu doping. The self-limiting doping process guarantees a high density of accommodation sites available for Al^3+^ and simultaneously facilitates their intercalation. Hereby, a specific discharge capacity of ~170 mAh g^−1^ at a current density of 25 mA g^−1^ is reached, leading to an energy density of 74 Wh kg^−1^. Furthermore, a Coulomb efficiency of almost 100% is achieved over 300 cycles at an even higher current density. Overall, our findings provide valuable insights on how to overcome current challenges in the development of AIBs. The demonstrated approach may be transferable to the fabrication of other batteries that operate with multivalent cations.

## Supplementary Materials

The following are available online at https://www.mdpi.com/2079-4991/10/2/247/s1, Figure S1: XRD pattern of pristine and cycled samples, Figure S2: Paper stability in the used ionic liquid-based electrolyte, Figure 3: Ex-situ TEM images after two CV cycles, Figure 4: Ex-situ TEM/EDX investigation after eight CV cycles, Table S1: Quantitative EDX analysis after eight CV cycles, Figure S5: Electron diffraction pattern, Figure S6: Scheme of the used cell setup, Figure S7: Stability test of tantalum in the electrolyte and voltage window, Figure S8: CV scans for bare Cu as cathode, Figure S9: Comparison of V_2_O_5_/Cu and Cu as cathodes, Figure S10: Galvanostatic charge/discharge test for bare Cu as cathode, Figure S11: Investigation of the pre-cycling by galvanostatic charge/discharge tests. Figure S12: Resistance vs. cycle number of the pre-cycling, Table S2: Comparison of various active materials in respect to their capacity and energy density.
