# Supplementary material for: Binder-Free V2O5 Cathode for High Energy Density Rechargeable Aluminum-Ion Batteries
Source: Nanomaterials (Basel). 2020 Jan 30;10(2):247. doi: 10.3390/nano10020247 (PMC7075190; doi:10.3390/nano10020247)
Supplement: Supplementary file 1 [file nanomaterials-10-00247-s001.pdf]

nanomaterials

Supporting Information

Binder-free V_2O_5 Cathode for High Energy Density Rechargeable Aluminum-ion Batteries

Achim M. Diem[1], Bernhard Fenk[2], Joachim Bill[1] and Zaklina Burghard[1,*]

[1] Institute for Materials Science, University of Stuttgart, Heisenbergstraße 3, 70569 Stuttgart, Germany;
[2] Max-Planck-Institute for Solid State Research, Heisenbergstraße 1, 70569 Stuttgart, Germany;
* Correspondence: zaklina.burghard@imw.uni-stuttgart.de; Tel.: +49-711-685-61958

Received: date; Accepted: date; Published: date

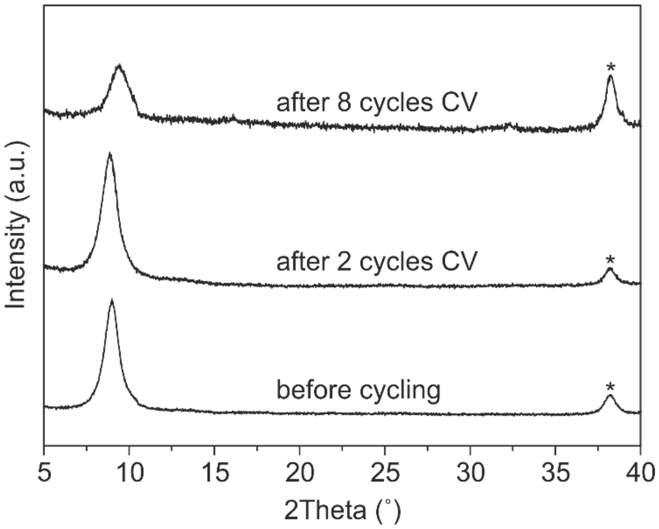

Figure S1: XRD patterns before and after electrochemical cycling (two and eight cycles CV). The intensity is normalized to one Au reflection (*).

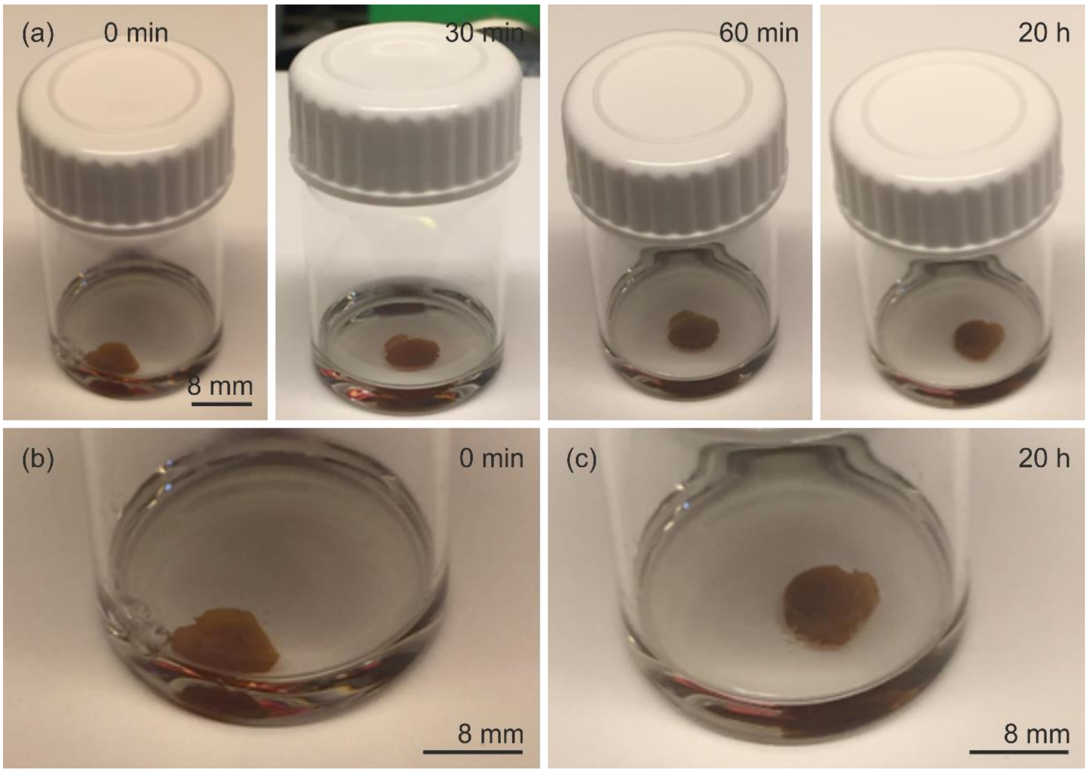

Figure S2. Digital images of the V_2O_5 paper like electrode immersed in the ionic liquid-based electrolyte after indicated time, demonstrating the electrode's chemical stability.

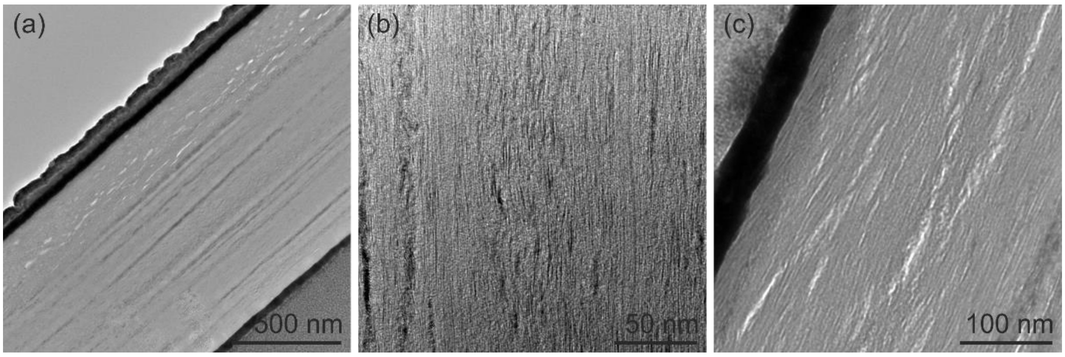

Figure S3: Ex-situ TEM images of the cross-section of a cathode, which was cycled for two cycles CV.

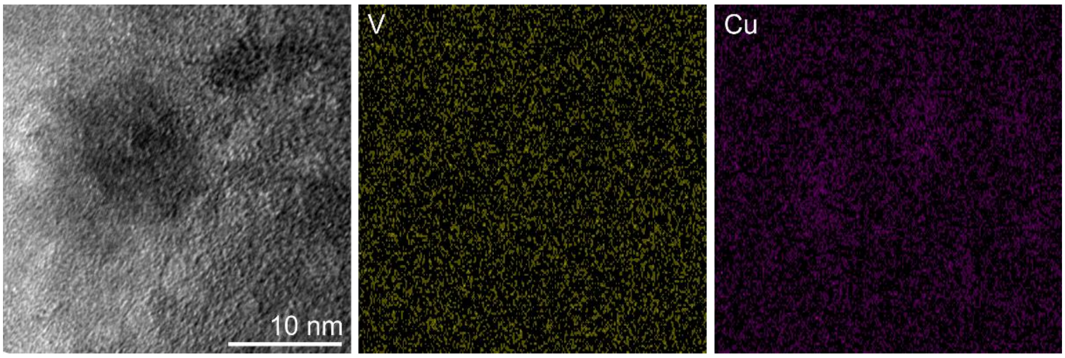

Figure S4: Ex-situ TEM investigation coupled with elemental mapping of the cross-section of a cathode, which was cycled for eight cycles CV.

Table S1: Ex-situ EDX analysis of a V_2O_5 cathode after 8 cycles of CV

	O (at%)	Al (at%)	Cu (at%)	V (at%)
**Matrix**	41.3	0.4	28.4	29.9
**Precipitate**	30.2	0.3	40.8	28.7

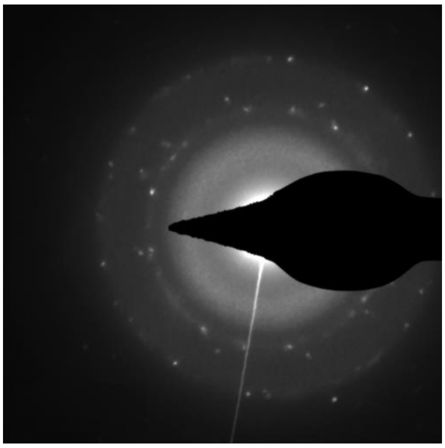

Figure S5: Electron diffraction reveals a ring-like diffraction pattern indicating a polycrystalline structure of our V_2O_5/Cu cathode. In particular, the (001) orientation of V_2O_5 can be observed. The residual diffraction rings might be referred to the Cu doped V_2O_5 and the nanocrystalline Cu-enriched precipitates.

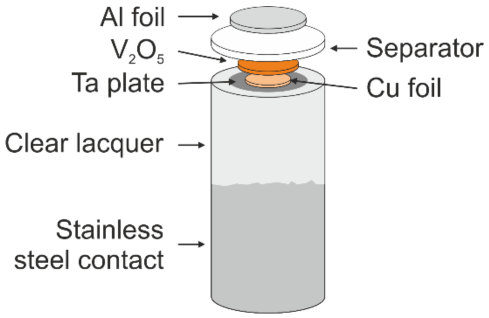

Figure S6: Scheme of the cell setup used for the electrochemical characterization of V_2O_5/Cu. The setup includes fixing a Ta plate on the stainless steel contact. The remaining stainless steel parts are coated with clear lacquer to avoid contact with the electrolyte.

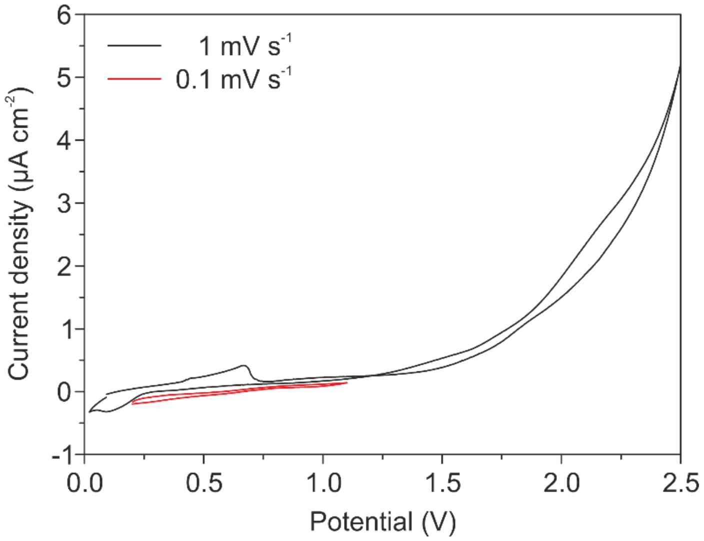

Figure S7: Stability test with Ta *vs.* Ta plate configuration, separated by one layer of glass fiber membrane. CV measurement in the potential window of 0.02-2.5 V and a scan rate of 1 mV s^{-1} (black line) revealing side reaction from ~1.5 V on. CV in the potential range of 0.2-1.1 V and a scan rate of 0.1 mV s^{-1} (red line) exhibiting no side reactions.

CV and galvanostatic charge/discharge measurements with bare Cu as cathode were performed to prove that the Cu is not electrochemically active. The visible peaks of the first CV cycle (**Figure S8**) reveal the dissolution of the native Cu oxide layer. From the cycle three on, only the anodic peak can be seen, which strongly decreases by further cycling. This indicates that the pure copper is dissolved by electrolyte and might be complexed by the chloroaluminate anions in the electrolyte. For the cycle five, almost no peaks are visible, revealing that the dissolution process is slow down and the saturation of Cu in the electrolyte is reached. Furthermore, the comparison of the CV curves of V_2O_5/Cu and bare Cu as cathode (**Figure S9)** reveals that the CV curve of V_2O_5/Cu shows distinct anodic and cathodic peaks, while the Cu dissolution is only an underlying reaction. This clearly excludes that the Cu shows electrochemical activity. Therefore, we prove that the electrochemical activity is attributed only to the intercalation of Al^{3+} and the redox reactions of V_2O_5. This was further verified by glavanostatic charge/discharge tests, showing that Cu only contributes in a negligible amount to the storage capacity (**Figure S10**). Moreover, the electrochemical activity of V_2O_5 towards the reversible intercalation of Al^{3+} was confirmed by Gu and coworkers.[1]

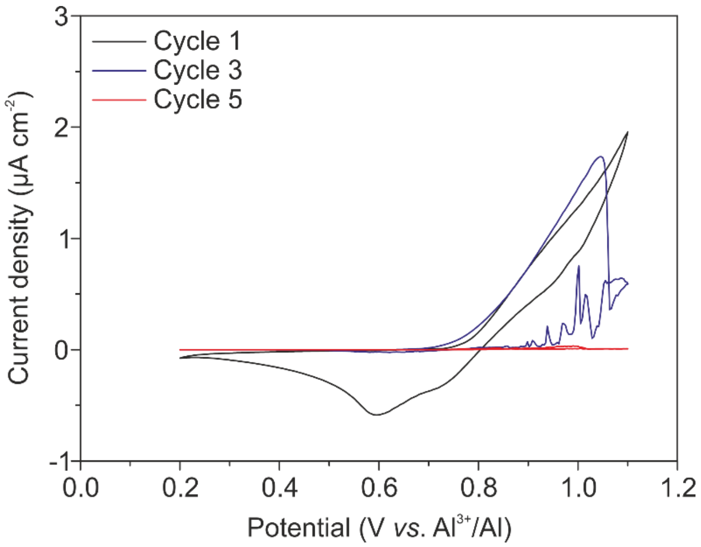

Figure S8: CV curves for a bare Cu cathode at a scan rate of 0.1 mV s^{-1}.

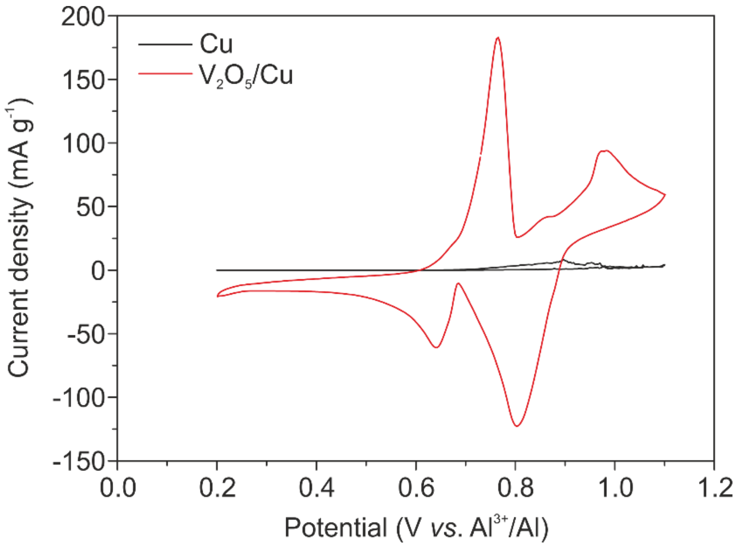

Figure S9: Comparison of CV curves of the 4th cycle for V_2O_5/Cu and bare Cu as cathode showing that the Cu dissolution is an underlying process.

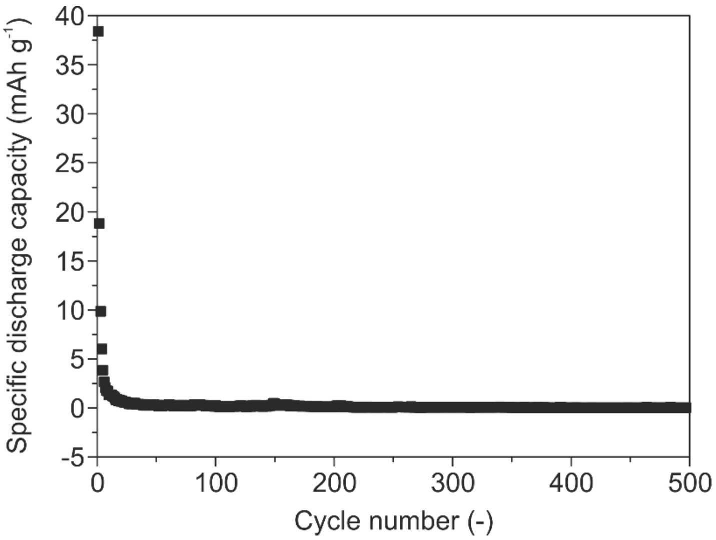

Figure S10: Specific discharge capacity obtained from galvanostatic charge/discharge tests with a current density of 100 mA g^{-1} of a bare Cu cathode vs. Al/Al^{3+}.

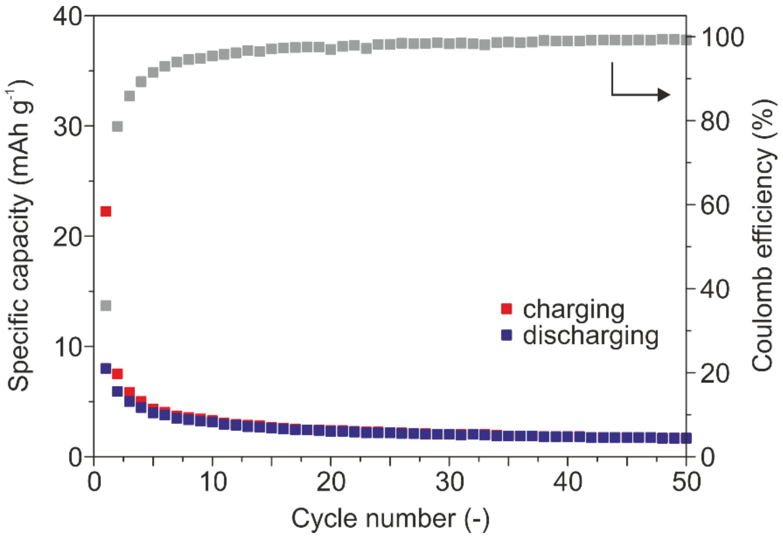

Figure S11: Storage capacity and Coulomb efficiency of the pre-cycling for 50 cycles at a current density of 1000 mA g^{-1}.

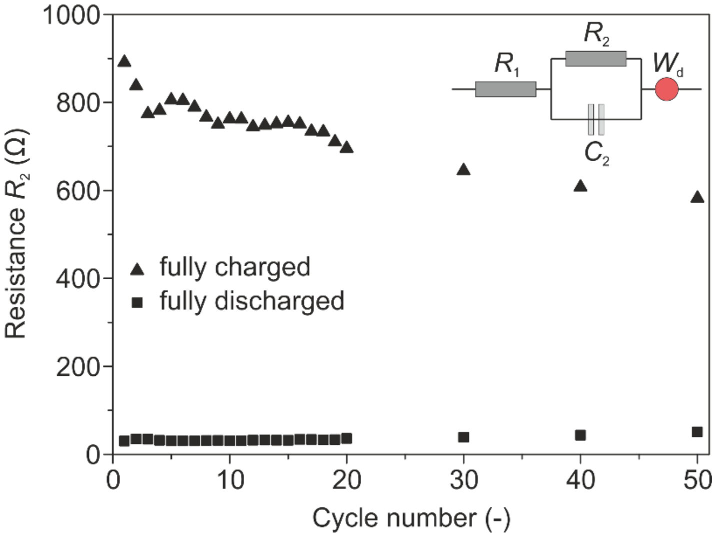

Figure S12: Investigation of the pre-cycling for 50 cycles at a current density of 1000 mA g^{-1}. At fully charged and discharged states EIS measurements were performed, which are fitted with the presented equivalent circuit model. R_1 represents the contact resistance, R_2 the bulk resistance, C_2 the bulk capacitance and W_d the Warburg element.

Table S2: Comparison of active material ratio, specific storage capacity, intercalation plateaus, current density and energy density for different electrode materials.

Electrode material	Amount of active material (%)	Specific discharge capacity ($mAh\ g^{-1}$)	Intercalation plateaus (V)	Current density ($mA\ g^{-1}$)	Energy density ($Wh\ kg^{-1}$)
**Carbon-based**					
**Smajic et al., Small, 2018.[2]**	80	171		100	170
**Lin et al., Nature, 2015.[3]**	100	65	2.25-2.0 & 1.9-1.5	n.a.	40
**Wang et al., Nature Communications, 2016.[4]**	90	110	2.25-2.0 & 1.9-1.5	99	69
**Zhang et al., Energy Storage Materials, 2018.[5]**	100	106	2.2-2.0 & 1.7-1.5*	4000	43***
**V_2O_5-based**					
**Wang et al., ACS Applied Materials & Interfaces, 2014.[6]**	100	239	0.7-0.5*	44	48***
**Chiku et al., ACS Applied Materials & Interfaces, 2015.[7]**	90	150*	1.0-0.7*	5	45***
**Gu et al., Energy Storage Materials, 2017[1]**	90	107	0.75-0.5*	n.a.	27***
**Wang et al., Advanced Energy Materials, 2017[8]**	n.a.	32*	0.5-0.3*	100	6***
**Wang et al., Journal of Materials Chemistry A, 2015.[9]**	80	100*	0.5-0.4**	10	10***
**Jayaprakash et al., Chemical Communications, 2011[10]**	85	273	0.6-0.5*	125	12***
**This work**					
**p-V_2O_5**	100	2.3	0.87-0.75 & 0.58-0.48	25	0.37
**V_2O_5/Cu**	100	173	0.86-0.77 & 0.68-0.59	25	74

Remarks:

*estimated for the 1st cycle

**estimated for the 2nd cycle

***calculation is based on provided storage capacity and insertion potentials

References

1. Gu, S.; Wang, H.; Wu, C.; Bai, Y.; Li, H.; Wu, F. Confirming reversible Al3+ storage mechanism through intercalation of Al3+ into V2O5 nanowires in a rechargeable aluminum battery. Energy Storage Mater. 2017, 6, 9–17.
2. Smajic, J.; Alazmi, A.; Batra, N.; Palanisamy, T.; Anjum, D.H.; Costa, P.M.F.J. Mesoporous Reduced Graphene Oxide as a High Capacity Cathode for Aluminum Batteries. Small 2018, 14, 1803584.
3. Lin, M.-C.; Gong, M.; Lu, B.; Wu, Y.; Wang, D.-Y.; Guan, M.; Angell, M.; Chen, C.; Yang, J.; Hwang, B.-J.; et al. An ultrafast rechargeable aluminium-ion battery. Nature 2015, 520, 324–328.
4. Wang, D.-Y.; Wei, C.-Y.; Lin, M.-C.; Pan, C.-J.; Chou, H.-L.; Chen, H.-A.; Gong, M.; Wu, Y.; Yuan, C.; Angell, M.; et al. Advanced rechargeable aluminium ion battery with a high-quality natural graphite cathode. Nat. Commun. 2017, 8, 14283.
5. Zhang, Q.; Wang, L.; Wang, J.; Xing, C.; Ge, J.; Fan, L.; Liu, Z.; Lu, X.; Wu, M.; Yu, X.; et al. Low-temperature synthesis of edge-rich graphene paper for high-performance aluminum batteries. Energy Storage Mater. 2018, 15, 361–367.
6. Wang, H.; Bai, Y.; Chen, S.; Luo, X.; Wu, C.; Wu, F.; Lu, J.; Amine, K. Binder-Free V2O5 Cathode for Greener Rechargeable Aluminum Battery. ACS Appl. Mater. Interfaces 2015, 7, 80–84.
7. Chiku, M.; Takeda, H.; Matsumura, S.; Higuchi, E.; Inoue, H. Amorphous Vanadium Oxide/Carbon Composite Positive Electrode for Rechargeable Aluminum Battery. ACS Appl. Mater. Interfaces 2015, 7, 24385–24389.
8. Wang, H.; Bi, X.; Bai, Y.; Wu, C.; Gu, S.; Chen, S.; Wu, F.; Amine, K.; Lu, J. Open-Structured V2O5·nH2O Nanoflakes as Highly Reversible Cathode Material for Monovalent and Multivalent Intercalation Batteries. Adv. Energy Mater. 2017, 7, 1602720.
9. Wang, H.; Gu, S.; Bai, Y.; Chen, S.; Zhu, N.; Wu, C.; Wu, F. Anion-effects on electrochemical properties of ionic liquid electrolytes for rechargeable aluminum batteries. J. Mater. Chem. A 2015, 3, 22677–22686.
10. Jayaprakash, N.; K. Das, S.; A. Archer, L. The rechargeable aluminum-ion battery. Chem. Commun. 2011, 47, 12610–12612.
